# Double Iatrogenic Esophageal and Duodenal Injury Induced by Endoscopic Retrograde Cholangiopancreatography: A Case Report

**DOI:** 10.7759/cureus.71355

**Published:** 2024-10-13

**Authors:** Shoag J Albugami, Nada N Binkhashlan, Rema F AlRashed, Faisal Alnefaie, Feras Alsannaa

**Affiliations:** 1 General Surgery, Prince Sultan Military Medical City, Riyadh, SAU; 2 Trauma and Acute Care Surgery, Prince Sultan Military Medical City, Riyadh, SAU

**Keywords:** duodenal injury, endoscopic retrograde cholangiopancreatography, endoscopy, esophageal injury, iatrogenic

## Abstract

Endoscopic retrograde cholangiopancreatography (ERCP) is a frequently performed procedure in the management of hepatobiliary diseases that can be conducted as a therapeutic or diagnostic procedure. Also, it can be done with or without sphincterotomy and stent insertion. Hemorrhage is one of the most common post-ERCP complications, which can be presented as late as 10 days. Other complications include post-ERCP pancreatitis and perforation. Gut perforation during ERCP is rare but often lethal. Here we present a 35-year-old female who was admitted to the hospital through the ER as a case of obstructive jaundice with common bile duct (CBD) stone. ERCP with stent insertion was performed for the patient to relieve the obstruction; however, intra-procedural retroperitoneal perforation was encountered.

## Introduction

Gut perforation during endoscopic retrograde cholangiopancreatography (ERCP) is rare but often lethal. In regular endoscopic procedures, iatrogenic gastrointestinal perforations occur at a rate of 0.03-0.8% [1], with a high mortality rate of 16% to 18% [2-5]. ERCP is a valuable procedure for diagnosing and treating pancreaticobiliary disorders, and it is a vital adjunct to imaging modalities such as magnetic resonance cholangiopancreatography or endoscopic ultrasonography. As for the etiology of ERCP perforations, previous studies showed that endoscopic sphincterotomy was responsible for 41%, insertion and manipulations of the endoscope were responsible for 26%, guidewires 15%, dilation of strictures 3%, other instruments 4%, stent insertion or migration 2%, and in 7% of cases the etiology was unknown [6-7]. Stapfer classification divides ERCP-related perforations into four types based on the mechanism, anatomical location, and severity of injury: type I comprising lateral or medial wall duodenal perforation; type II comprising perivaterian injuries; type III comprising distal bile duct injuries due to guidewire-basket equipment; and type IV comprising retroperitoneal air alone. ERCP-related perforations can usually be diagnosed during ERCP, from the endoscopic view or using fluoroscopy [6]. For management, it is reported in the literature that unless endoscopic closure can be achieved, type I perforations require immediate surgical repair. Patients with type II perforations should be treated non-operatively at first. Non-surgical therapy options include biliary stenting, fasting, intravenous fluid resuscitation, nasogastric drainage, broad-spectrum antibiotics, percutaneous fluid collection drainage, and total parenteral nutrition (TPN) if long periods of fasting are suspected. Non-operative treatment was successful in 79% of type II injury patients. In all patients with type III injuries, non-operative therapy was adequate [6]. Here we present a case of a 35-year-old female who underwent ERCP that was complicated with duodenal perforation.

## Case presentation

A 35-year-old female who did not have any comorbidity was not using any medication and had never undergone any surgery came to the ER complaining of persistent right upper quadrant pain for one day, not associated with nausea, vomiting, or change in urine or stool. There was no history of fever or night sweats, but she had a previous similar pain attack for one month relieved by analgesics. On examination, the patient was vitally stable, afebrile, with no tachycardia, and had good oxygen saturation on room air. She was not in severe pain, not jaundiced, and her abdomen was soft and lax with right upper quadrant tenderness. Her laboratory investigation results are presented in Table 1.

**Table 1:** Laboratory investigation upon initial presentation INR: international normalized ratio

Laboratory investigation	Result	Reference values
White blood cell (WBC)	5.6 × 10^9^/L	4.0-11.0 × 10^9^/L
Hemoglobin (Hgb)	12 g/dl	11.5-16.5 g/dl
Platelets	289 × 10^9^/L	150-450 × 10^9^/L
INR	0.9	0.9-1.3
Creatinine	80 μmol/L	45-84 μmol/L
Total bilirubin	19.5 μmol/L	2-21 μmol/L
Direct bilirubin	12.2 μmol/L	1-5 μmol/L
Alanine transaminase (ALT)	400 U/L	4-36 U/L
Alkaline phosphatase (ALP)	113 U/L	35-104 U/L
Potassium	3.8 mEq/L	3.5-5.1 mEq/L
Sodium	137 mEq/L	136-145 mEq/L

Abdomen ultrasound showed that the gallbladder contained a 0.4 cm stone with no signs of cholecystitis. The liver showed homogenous echotexture with a smooth outline. The common bile duct (CBD) measured 0.5 cm with a lower CBD 0.4 cm stone noted without biliary ductal dilatation.

The patient was admitted with a case of obstructive jaundice and kept nothing per oral (NPO) on intravenous fluids, pain control, started on gastrointestinal tract and venous thromboembolism (VTE) prophylaxis, and antibiotics. A gastroenterology physician was consulted, and they recommended ERCP the next day. During ERCP, sphincterotomy was done with difficulty, no pancreatic duct (PD) cannulation was done, a large CBD stone was retrieved, and a 7 French stent was placed. Intra-procedural retroperitoneal perforation was encountered. The patient was assessed post-ERCP, and she was vitally stable, had no tachycardia, was alert and oriented, and complained of shortness of breath. The abdomen was soft and lax with no peritoneal signs, no guarding, or rigidity. Upon chest examination, there was an absent air sound on the right side.

**Figure 1:**
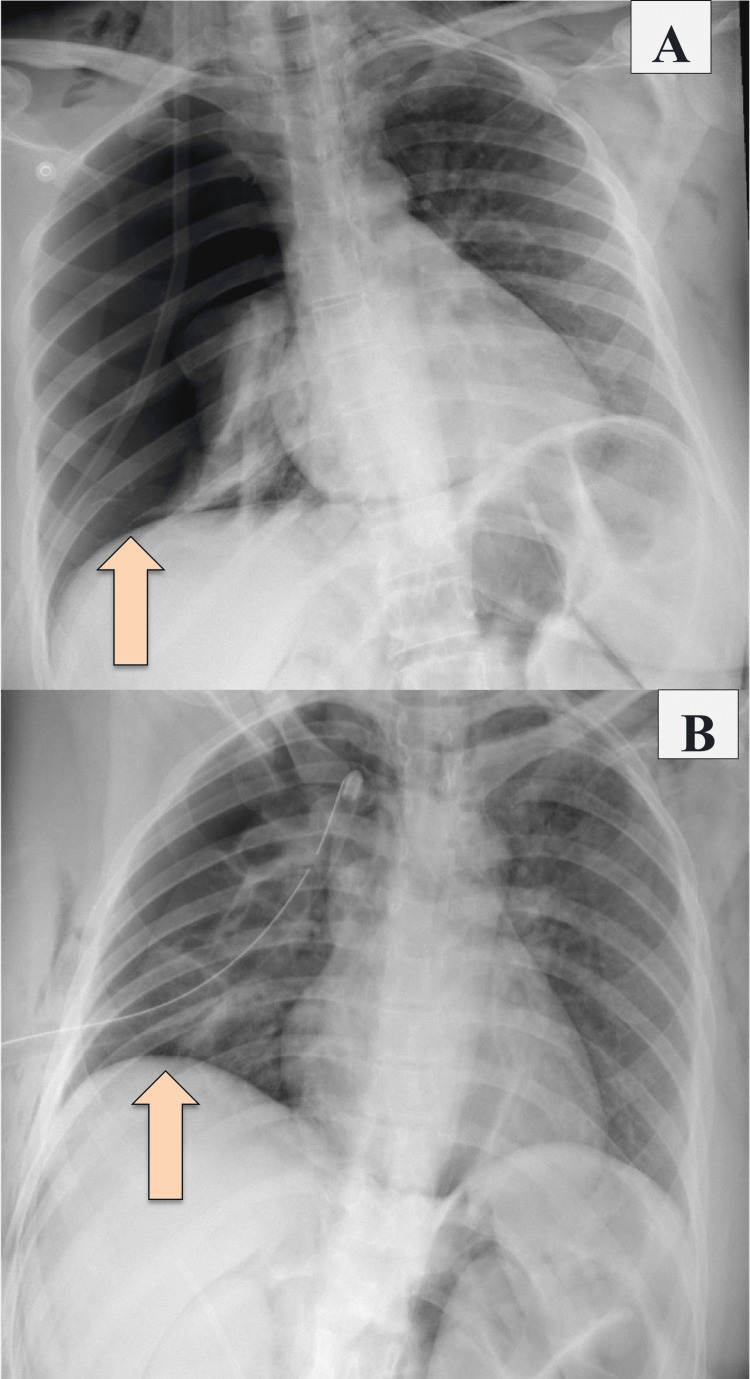
Chest X-ray showing right pneumothorax and pneumediastinum pre- and post-chest tube insertion A: Before chest tube insertion (the arrow is pointing at the pneumothorax before tube insertion); B: After chest tube insertion (the arrow is pointing at the resolution of pneumothorax after tube insertion)

A nasogastric tube (NGT) was inserted and kept on free drainage (no bilious content was seen). She was kept NPO on IV fluid, serial abdominal examinations were done, and the patient was planned for CT abdomen with IV and oral contrast. A chest X-ray was done and showed right-sided pneumothorax and pneumomediastinum, and for that, a chest tube was inserted (Figure 1).

**Figure 2:**
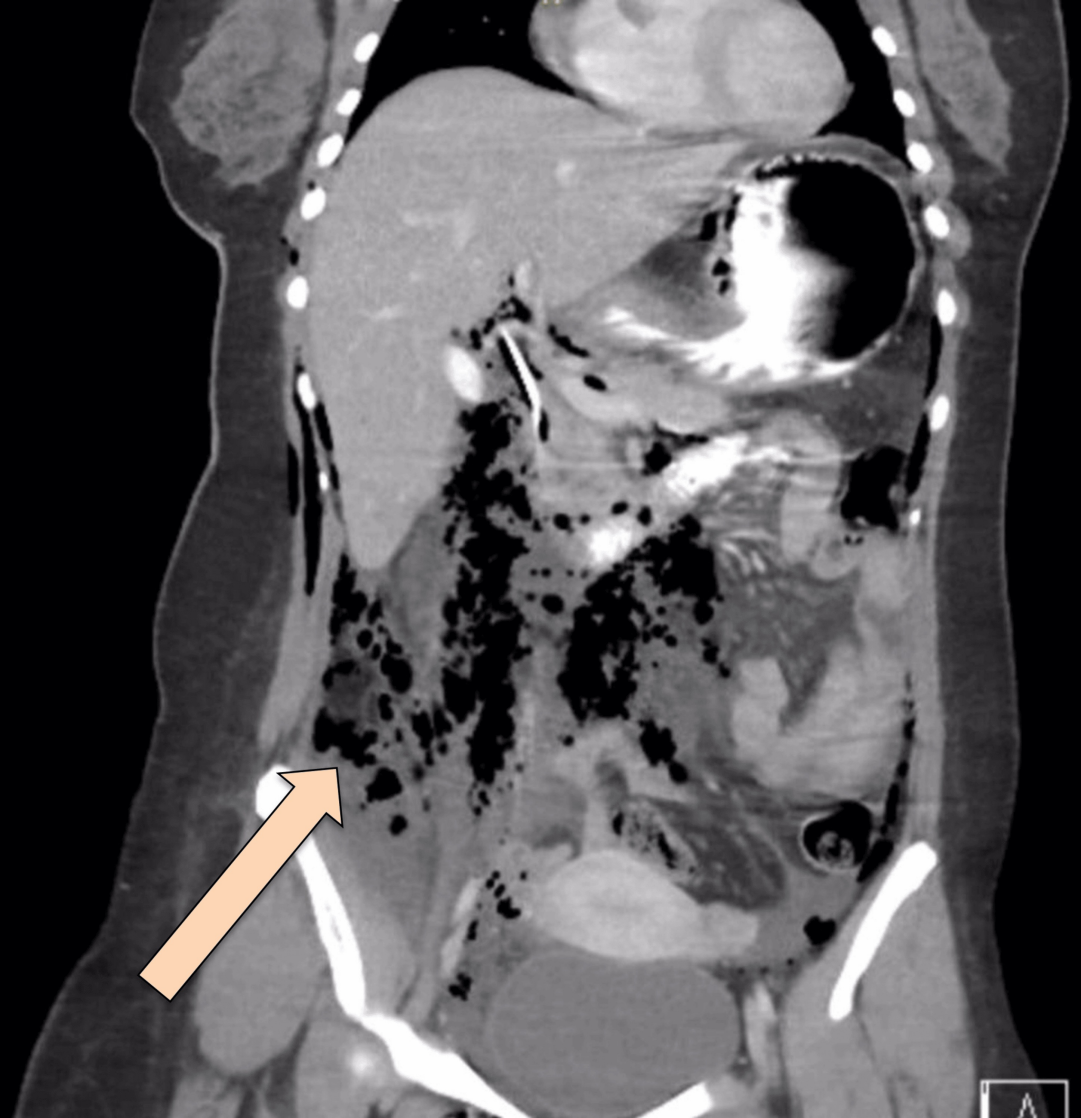
Coronal view of CT abdomen with IV and oral contrast post-ERCP showing extensive pneumoperitoneum The arrow is pointing at the extensive pneumoperitoneum CT: computed tomography; IV: intravenous; ERCP: endoscopic retrograde cholangiopancreatography

**Figure 3:**
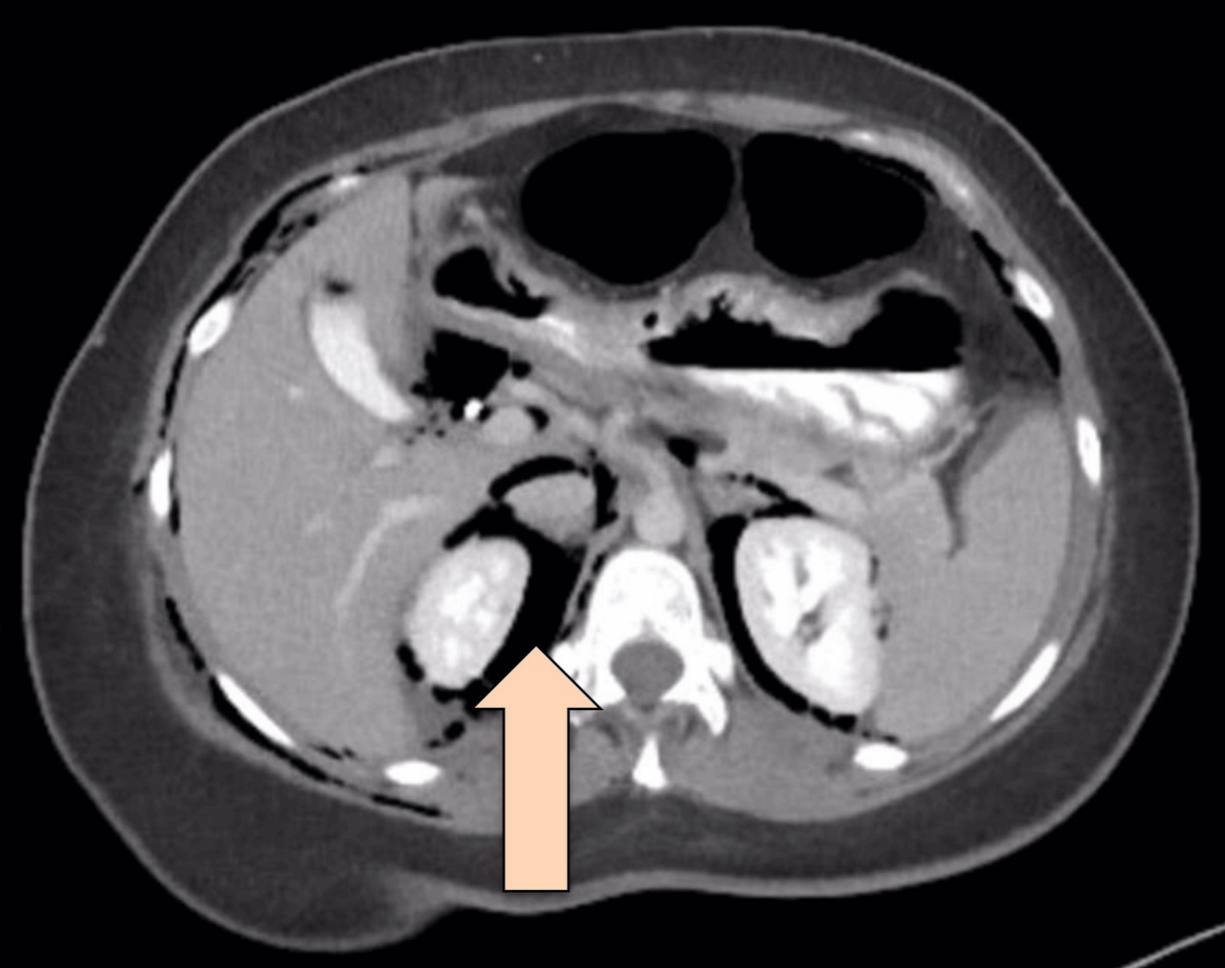
Axial view of CT abdomen with IV and oral contrast post-ERCP showing extensive pneumoperitoneum The arrow is pointing at the extensive pneumoperitoneum CT: computed tomography; IV: intravenous; ERCP: endoscopic retrograde cholangiopancreatography

**Figure 4:**
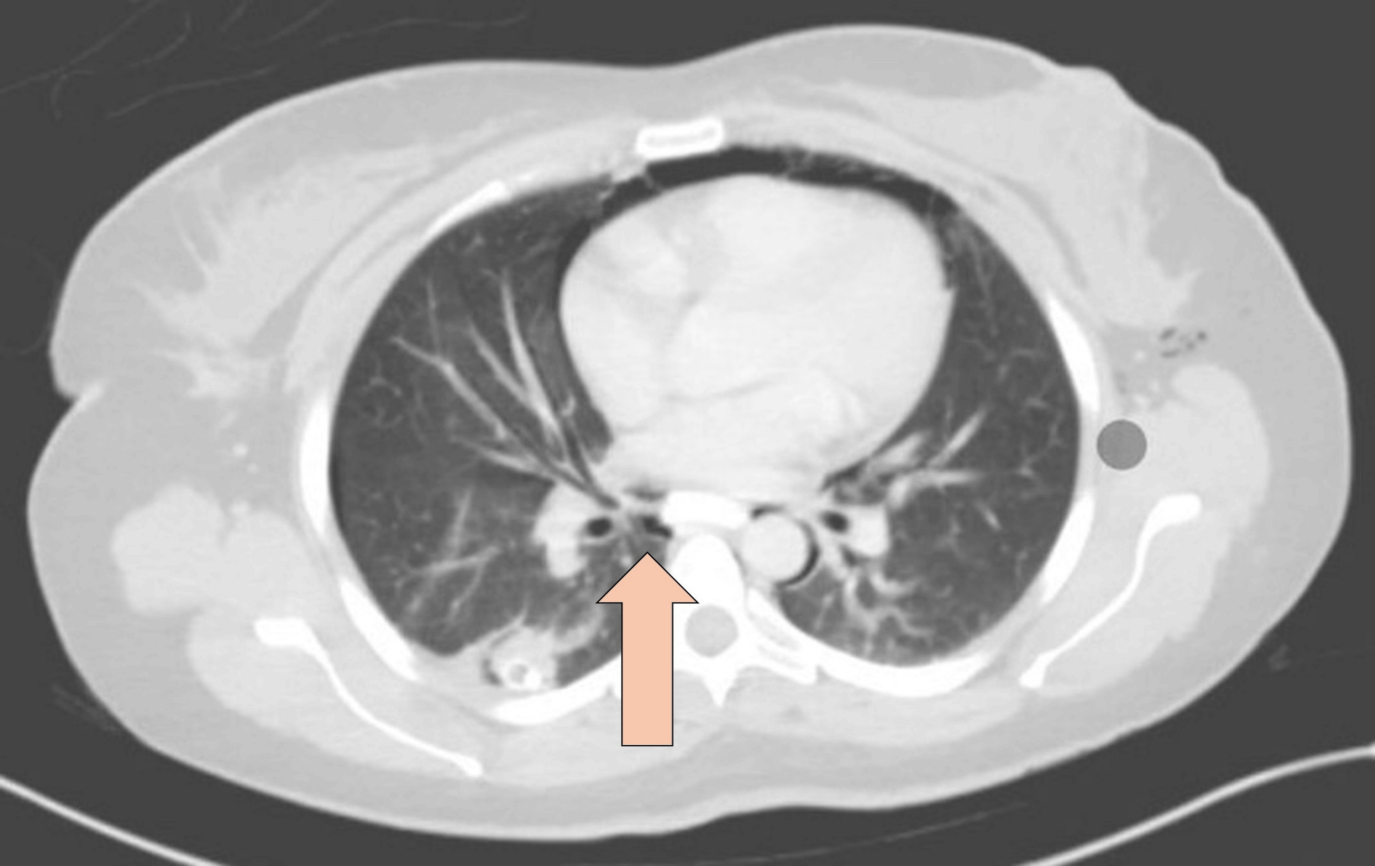
Axial view of CT abdomen with IV and oral contrast post-ERCP showing esophageal perforation The arrow is pointing at the site of esophageal perforation CT: computed tomography; IV: intravenous; ERCP: endoscopic retrograde cholangiopancreatography

CT abdomen with IV and oral contrast post-ERCP showed pneumobilia with evidence of stent in the biliary system seen in a satisfactory position, significant pneumoretroperitoneam, small amount of retroperitoneal leaked contrast inferior to the second part of the duodenum, bulky and edematous pancreatic head, and uncinate process suggestive of acute pancreatitis, no intra-abdominal collection, and minimal amount of free fluids. There was extensive soft tissue emphysema along the anterior neck, lateral chest, and abdominal wall, severe pneumomediastinum, and minimal bilateral pneumothoraxes, with a chest tube seen on the right side in a satisfactory position, and a tiny area of contrast leakage was seen in the right posterior-lateral of the mid esophagus at T6 (Figures 2-4). The impression was double perforation at the second part of the duodenum and esophagus.

The patient was reassessed and monitored closely; her heart rate was 130 beats per minute (BPM), she was afebrile, her oxygen saturation was 94% on a 2-liter nasal cannula, and her blood pressure was maintained. She was kept NPO, the NG tube was in place, the patient was on IV fluid, and was shifted to the ICU for close monitoring. The patient was started on tazocin and subcutaneous octreotide. Thoracic consultation for esophageal perforation was obtained, and they recommended keeping the NG tube on low intermittent suction and an upper gastrointestinal (UGI) study after 72 hours to assess the esophagus. The UGI study was done using a gastrograffin, and there was free flow of contrast in the esophagus without evidence of filling defects, contrast extravasation, or stricture, and no gross leak of contrast was seen from the duodenum.

The patient was started on TPN and kept NPO. US/fluoroscopic guided drainage was done for the retroperitoneal collection; a drain was inserted in the right lower quadrant (RLQ) with serosanguinous drainage. The patient was assessed post-drainage; she was vitally stable apart from tachycardia but improving, and the abdomen was soft and lax with no peritoneal signs. Despite that, the patient had a persistently high WBC count, reaching 19 × 10^9^/L. For which tigecycline was started empirically.

**Figure 5:**
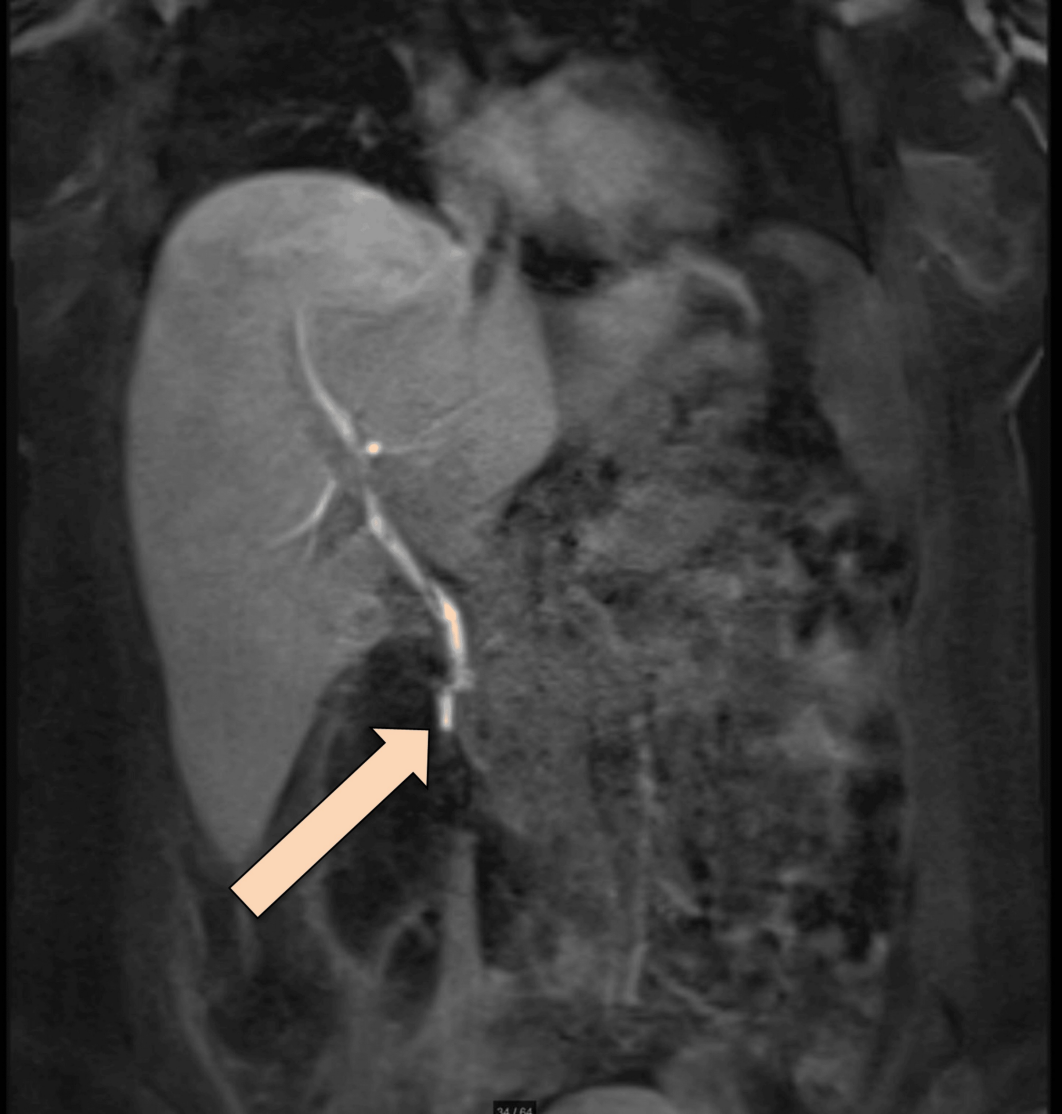
MRCP Promovist The arrow is pointing at the injured site in the distal CBD MRCP: magnetic resonance cholangiopancreatography; CBD: common bile duct

After the lungs were fully expanded in the chest X-ray, the chest tube was removed. The plan was to proceed with an MRCP Promovist to assess for a persistent biliary leak. Pooling/extravasation of Primovist into the retroperitoneal collection was observed, with the injury site likely at distal CBD at the level of the ampulla of Vater. The stable size of the multiloculated air-containing retroperitoneal collection was noted, with features suggesting a superadded infectious process (Figure 5).

**Figure 6:**
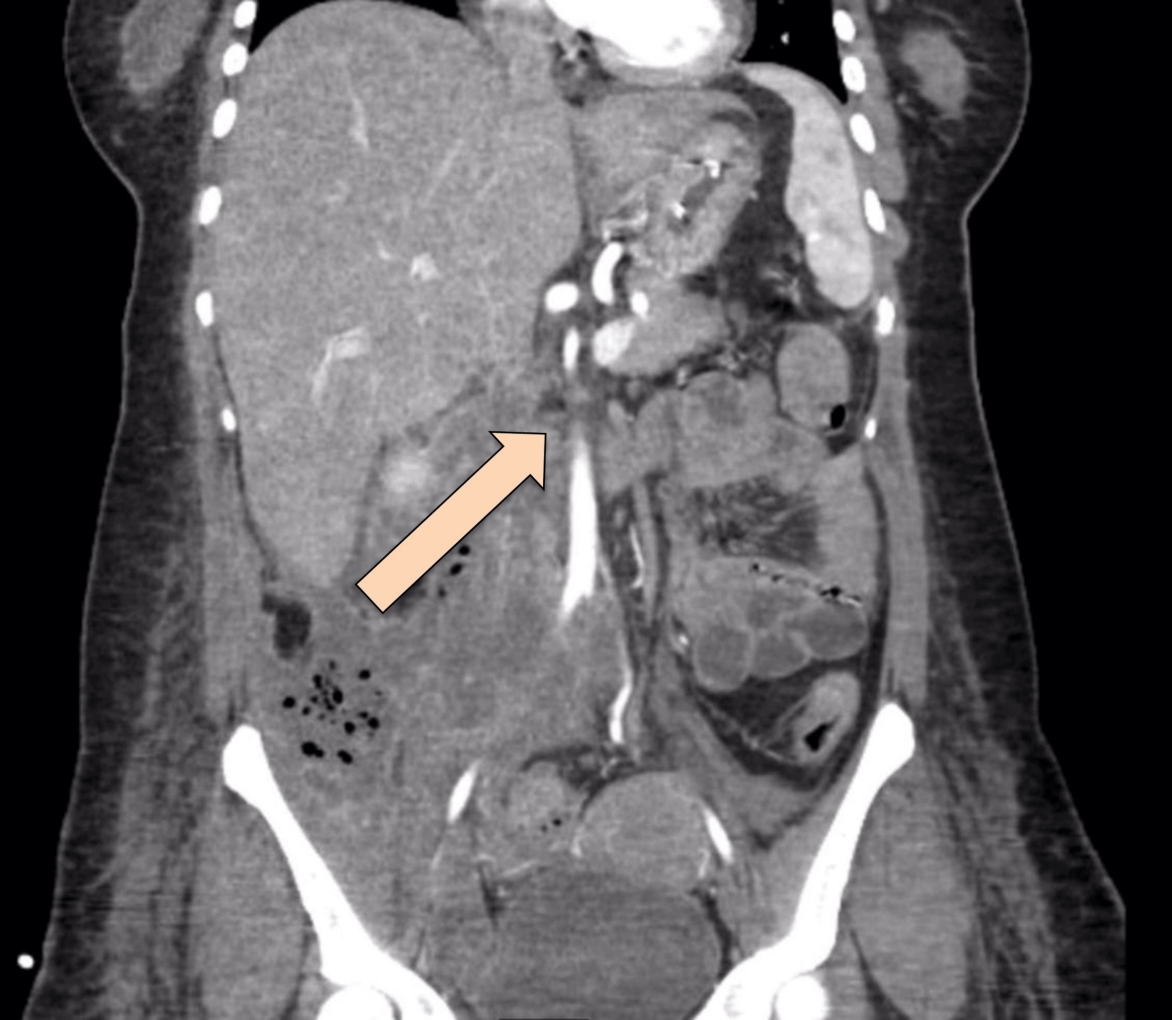
Follow-up coronal view of CT abdomen with IV contrast The arrow shows persistent communication between the para-duodenal collection and the posterior aspect of the distal CBD CT: computed tomography; IV: intravenous; CBD: common bile duct

**Figure 7:**
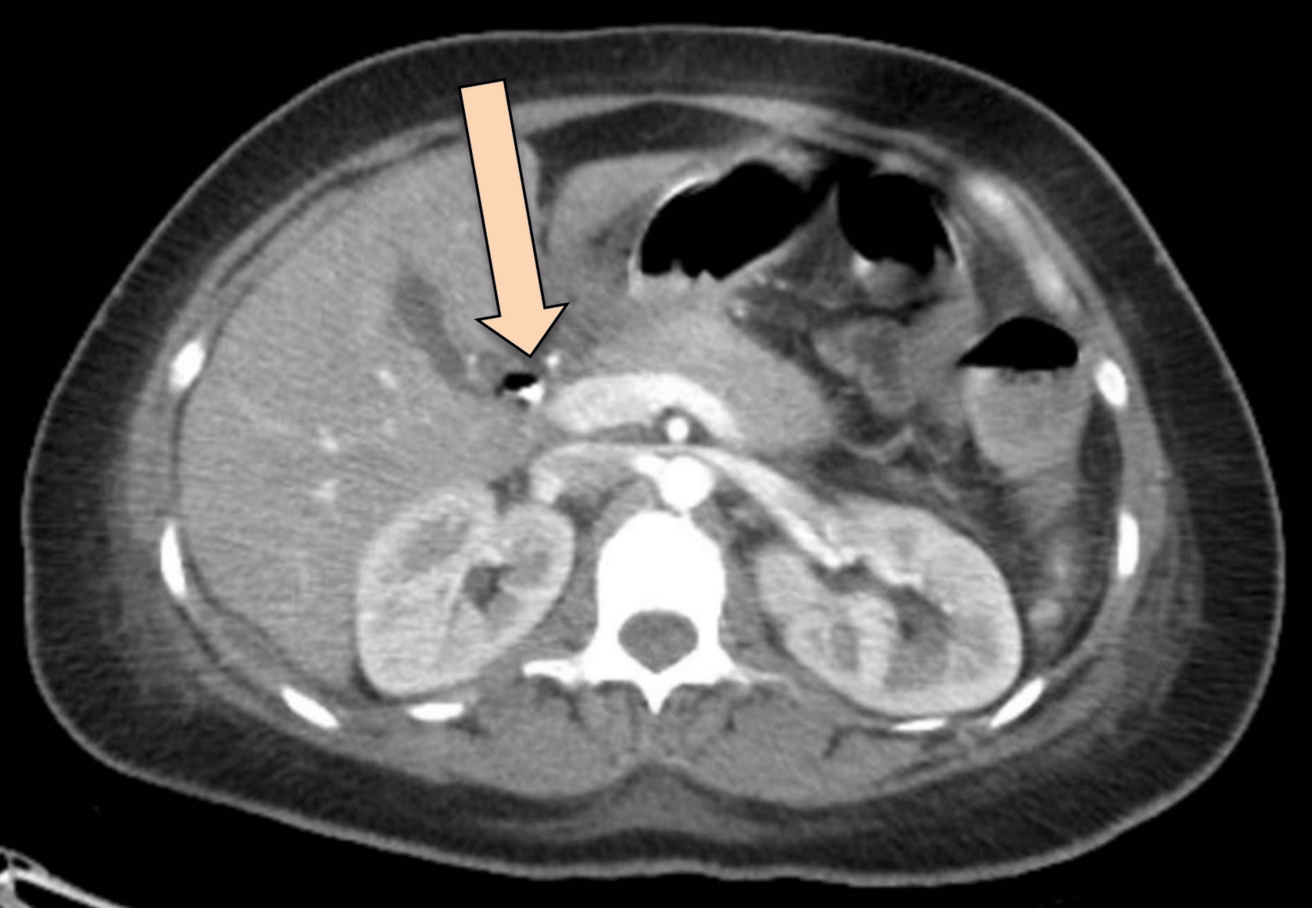
Follow-up axial view CT abdomen with IV contrast The arrow shows persistent communication between the para-duodenal collection and the posterior aspect of the distal CBD CT: computed tomography; IV: intravenous; CBD: common bile duct

Since the patient was still tachycardic and still had leukocytosis, interventional radiology (IR) was consulted on upsizing the collection drainage to 12 French catheters. Drain output was around 100-150 ml/24 hours, billous, and started to be mixed with pus. Follow-up CT showed persistent communication of the retroperitoneal paraduodenal collection with the posterior aspect of the most distal part of the CBD. The oral contrast flow through the entire GI tract showed no evidence of a leak. Interval change in the position of the iliac fossa drain was observed, with its tip seen within the upper aspect of the right iliac fossa collection. A reduction in the size of the multi-loculated retroperitoneal collections was noted. The largest locule is seen in the right iliac fossa, which measures 9 x 4 x 12.8 cm, compared to 10 x 5 x 15 cm. Another locule was seen posterior to the duodenum, measuring 3.6 x 2.7 x 9.4 cm compared to 4.3 x 5.8 x 11 cm (Figures 6-7).

Under fluoroscopy guidance, another drainage catheter was inserted in the RLQ. The previous drainage catheter was replaced, and a total of 45 ml of purulent fluids was aspirated. The contrast was injected through a drain, which showed communication/reflux of contrast into CBD, which indicated persistent communication/leak.

The patient was clinically stable and responding to non-operative treatment. Vitally stable, heart rate was 90 BPM, temperature was 37°C, blood pressure was maintained with good oxygen saturation in room air, on TPN feeding, NGT draining 100-300 ml/24 hour. The output of the para-duodenal drain was draining approximately 15-30 ml of bile every 24 hours. The RLQ drain showed pus with an output of 60-100 ml/24 hours. Laboratory investigations showed the following: WBC of 7.3 × 10^9^/L, hemoglobin (Hgb) of 9.8 g/dl, platelets of 295 x 10^9^/L, INR of 1.2, total bilirubin of 13 μmol/L, direct bilirubin of 8 µmol/L, ALT of 21 U/L, albumin of 27 g/liter, urea and electrolyte were within normal levels, CRP of 35 mg/L, and procalcitonin of 0.60 μg/L.

NGT was clamped for two days, and the patient was given Methylene blue orally to rule out a leak. Drains were assessed with no change in color noticed with an output of brownish 12 ml/24 hour for the right upper drain and greenish turbid 62 ml/24 hour for the right lower drain.

Then NGT was removed, and the patient was started on a clear liquid diet, which was advanced to a regular diet as the patient tolerated. Total parental nutrition was stopped two days later.

Tigacylin was stopped by the infectious diseases team after approximately one month; the next day the patient was doing fine with no complaints, was tolerating orally and was hemodynamically stable. Although WBC increased from 8.9 to 11.6, renal profile and liver function tests were within normal limits, cultures from the drain were sent, and meropenem was started one week after stopping the tigacylcin.

Then the patient was symptomatic-free and hemodynamically stable except for tachycardia, as heart rate was 116 BPM, WBC decreased to 7.5 × 10^9^/L, Hgb was 9.8 g/dl, platelets were 369 x 10^9^/L, renal profile, and electrolytes were within her baseline.

**Figure 8:**
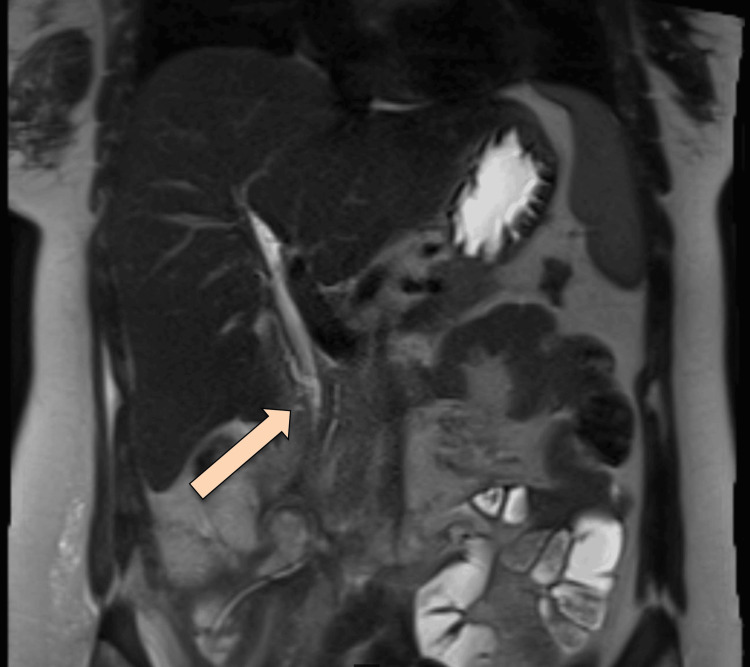
Follow-up MRCP The arrow is pointing at the biliary leak MRCP: magnetic resonance cholangio pancreatography

MRCP was repeated, which showed delayed phases of the hepatocellular agent demonstrating opacification of the biliary tree with contrast reaching the second part of the duodenum without evidence of contrast leakage. However, 12 hours of delayed images showed a faint high signal at the expected site of the collection, possibly representing a persistent biliary leak. No significant biliary duct dilatation was observed. Expected minimal pneumobilia was noted (Figure 8).

The cultures that were sent from the drains were positive for *Pseudomonas aeruginosa*; meropenem was stopped and ceftazidime was started.

The patient was seen on a daily basis; she was symptomatic-free, her vitals were within normal, and her creatinine, electrolyte, and liver function tests were within normal limits.

Ten weeks after admission, the patient was discharged; upon discharge, she was clinically stable with no complaint, tolerating orally, and passing a normal bowel motion. On physical examination, she was vitally stable with a temperature of 36.7°C, heart rate of 92 BPM, blood pressure of 104/63, oxygen saturation of 98% on room air, and her abdomen was soft and lax. Both drains output was 10 ml/24 hours, and one of the drains was removed upon discharge. Her laboratory investigations showed WBC of 8.2 × 10^9^/L, Hgb 10.9 g/dl, platelets of 395 x10^9^/L, total bilirubin of 18 μmol/L, direct bilirubin of 10 μmol/L, her renal profile and electrolytes were within normal limit, and CRP was 7 mg/L. The patient was seen in the clinic and was planned for elective admission for ERCP.

ERCP was done where the scope was advanced up to the second part of the duodenum, which showed the previous CBD stent, which was removed by grasper, and a clear mucosal defect was seen. Over-the-scope clips (OVESCO closing device) were used to close the penetrating mucosal defect, and a new CBD stent was inserted.

An upper GI study done two days later showed no contrast leak from the examined portions of the small bowel.

The patient was discharged the next day and had no complaints, was hemodynamically stable, was afebrile, and her abdomen was soft and lax with no tenderness.

## Discussion

ERCP is a commonly done procedure nowadays for hepatobiliary diseases, which can be done as a therapeutic or diagnostic procedure. It can be done with or without sphincterotomy and stent insertion. Hemorrhage is one of the most common post-ERCP complications (an incidence of 10%-30%) which can be presented as late as 10 days [6,8-10]. Other post-ERCP complications include pancreatitis and perforation, with an incidence of less than 1% to 40% and less than 1%, respectively, according to the patient’s and technique-related risk factors. Although perforation is a rare complication, it is associated with a mortality rate that can reach 26.9%. The severity of the previously mentioned complications can range from hospitalization for a few days to death with a rate of less than 0.5% [6,11-13].

Perforation can usually be identified during the procedure as a contrast extravasation, free gas seen in extraluminal retroperitoneal or intraperitoneal, or after the procedure as a deterioration of the patient’s clinical status post-procedure if the patient had fever, abdominal pain, hematemesis, hematochezia, leukocytosis, or peritoneal signs. Then early diagnosis by radiography and early management are advised [6].

This case report describes an uncommon case of double Iatrogenic esophageal and duodenal injury induced by ERCP, complicated by acute pancreatitis and significant pneumoperitoneum, which was successfully managed conservatively.

Conservative management included fluids, wide-spectrum antibiotics, adequate analgesia, and intravenous proton pump inhibitors. All patients undergo nasogastric or nasoduodenal aspiration to divert the luminal material, and complete parenteral feeding is required. CT-guided percutaneous drainage is considered the core management when intra-abdominal collections happen as it’s precise, safe, and effective. However, follow-up imaging is required to ensure that the collection size is regressing and that the tip of the catheter is not misplaced. In follow-up imaging for our case, multiple exchanges, either due to misplacement or blockage, where upsizing of the drain was done.

During follow-up, cross-sectional imaging should be carried out, and percutaneous drainage should be taken into consideration if a liquid collection is revealed. If there is no more leakage found by the fourth day, oral intake can be resumed [11]. However, in our case, we elected to keep the patient NPO as she has concurrent esophageal perforation. In cases where a duodenal wall perforation is discovered during the treatment or before the 12-hour mark, endoscopic or conservative measures may be taken [12]. In certain individuals who are in good condition and do not exhibit contrast medium extravasation or a persistent big fluid collection on CT scan, endoscopic therapy of the duodenum perforation may be carried out if it is discovered later (after more than 12 hours) [12]. Our patient had a large collection, for which we preferred drainage rather than endoscopic management. Stapfer et al. [7] in their classification declared that types 2 and 3 or type 4 injuries may be managed conservatively.

The majority of esophageal perforations occur in the thoracic portion of the esophagus and are linked to therapeutic endoscopic procedures [13-14]. The most common significant consequence of esophageal dilatation that is reported is iatrogenic perforation [13]. During our case, the patient developed esophageal perforation due to ERCP, where the area of maximum manipulation was the periampullary area, which can be attributed to over-insufflation, which was used during the ERCP to achieve a better view during difficult ERCP [15-16].

Delayed surgical management will be indicated once the patient remains septic and not controlled despite the non-operative management [6].

## Conclusions

Conservative management can be used in the case of double esophageal and duodenal iatrogenic perforation. General patient condition, site of perforation, and patient stability are factors that influence the choice of management. During follow-up, cross-sectional imaging should be carried out to evaluate the efficacy of conservative management. Also, percutaneous drainage should be taken into consideration if a liquid collection is revealed, and diet can be resumed if no more leak is present. Delayed surgical management is indicated if the patient remains in sepsis and is not controlled despite the non-operative management.
